# Postoperative Analgesia in Modified Radical Mastectomy Patients After Instillation of Bupivacaine Through Surgical Drains

**DOI:** 10.7759/cureus.24125

**Published:** 2022-04-13

**Authors:** Uzma Shamim Seth, Sughra Perveen, Tanweer Ahmed, Mohammad Taha Kamal, Jehangir Ali Soomro, Munira Murtaza Khomusi, Maha Kamal

**Affiliations:** 1 Surgical Ward 3, Unit 1, Jinnah Postgraduate Medical Centre, Karachi, PAK; 2 General Surgery, Jinnah Postgraduate Medical Centre, Karachi, PAK; 3 Thoracic Surgery, Shaheed Mohtarma Benazir Bhutto Institute of Trauma, Karachi, PAK; 4 Surgery, Jinnah Postgraduate Medical Centre, Karachi, PAK; 5 Surgical Ward 1, Jinnah Postgraduate Medical Centre, Karachi, PAK; 6 Medicine, Jinnah Postgraduate Medical Centre, Karachi, PAK

**Keywords:** modified radical mastectomy (mrm), surgical drains, bupivacaine, analgesia, breast cancer

## Abstract

Background: In contrast to other breast surgeries, modified radical mastectomy (MRM) with axillary lymph node clearance involves intense tissue dissection, with postoperative seroma formation and pain being the major complaints affecting patients. Among these, 40% of females experience acute postoperative pain, and between 25 to 60% develop persistent chronic postsurgical pain. The rationale of this study was that minimally invasive procedures can result in immediate pain relief in patients undergoing mastectomy, which has been proven to satisfy their needs and lead to early discharge in the local population.

Objective: This study determined to find out the efficacy of instilling bupivacaine on wounds by means of surgical drains in controlling pain after MRM.

Methodology: This was a randomized control study trial that was carried out in Surgical Unit 1, Ward 3, Jinnah Postgraduate Medical Centre, Karachi, from November 2020 to April 2021. All patients tested negative for coronavirus disease 2019 (COVID-19) by PCR test before randomly allocating them into two groups. Thirty women in Group B received 40 ml of 0.25% injection bupivacaine, and 30 in Group C received no drug. Duration of analgesia was recorded as time in hours when the patient was received after surgery in the post-anesthesia care unit until the patient felt ache and discomfort of > three scores according to the visual analog pain score chart (VAS).

Results: The average age was 52.48±4.76 years. The mean period of time during which analgesia was observed was significantly higher in Group B as compared to Group C (10.93±1.84 vs 5.03±1.35 hours, p=0.0005).

Conclusion: There is improvement in postoperative analgesia after instilling bupivacaine through surgical drains on wound beds in MRM patients.

## Introduction

Around the world, breast cancer is now acknowledged as the most prevalent cancer among females. The estimated incidence of breast cancer was 20% which has risen drastically since 2008, while mortality has escalated by 14%. One in every four females dies due to breast cancer [1]. In spite of improvements in medical treatment, surgery plays a principal role in its management. Unfortunately, very little advancement has been made to improve postoperative pain control after modified radical mastectomy (MRM). Today, about one in every two patients still complains of considerable postoperative pain after breast surgery [2]. MRM accounts for 31% of all breast surgeries [3]. In variation to other breast surgeries, MRM with axillary lymph node clearance involves intense tissue dissection, with postoperative seroma formation and pain being the major complaint affecting patients. Pain is one of the most commonly encountered symptoms in up to 50% of women who receive mastectomy [4].

Among these, 40% of the females experienced acute postoperative pain, and between 25 to 60% developed persistent chronic postsurgical pain [5,6]. The patient complains of burning, pressure sensation, and numbness confined to the anterior and lateral chest wall, upper limb, and axilla [7]. Tissue damage causes pain due to inflammation, and neuropathic pain results from disruption of the second to sixth intercostal nerves. This results in severe discomfort with longer hospital stays and increases in postsurgical admissions to the hospital [8]. Various pain-controlling methods are used to decrease post-surgical pain. Opioid side effects include nausea, vomiting, shallow breathing, sedation, and dizziness, whereas non-steroidal anti-inflammatory drugs (NSAIDs) are associated with indigestion and heartburn. In addition to these, interventional techniques include local anesthetic infiltration and wound infusion with liposome bupivacaine. Regional anesthesia includes intercostal blocks, paravertebral blocks, thoracic epidural anesthesia, intrapleural blocks, and ultrasound-guided interfascial plane blocks [9].

The rationale of this study was that minimally invasive procedures can result in immediate pain relief in patients undergoing mastectomy, which has been proven to satisfy their needs and early discharge in the local population. This will eliminate pain with the least side effects and at a minimal cost. The entire household chores of our community depend on females. If they have no discomfort after the operation, they will have a rapid recovery and return early to domestic responsibilities. This study will also help the medical community by reducing the number of admissions due to pain after mastectomy, early discharge, and reduction in the use of narcotics for pain relief such as morphine and fentanyl and their adverse effect on patients. The aim of this study is to determine the efficacy of bupivacaine instillation through surgical drains on a wound bed in controlling postoperative pain after MRM by comparing the mean duration of analgesia.

## Materials and methods

After informed consent, 60 female patients aged between 45 to 60 years with the American Society of Anesthesiologists (ASA) physical status classification system I or II (Appendix II), undergoing unilateral MRM with axillary lymph node dissection were involved in this study. This was a randomized controlled trial (RCT) study with a probability random sampling technique. This study was conducted in Surgical Unit 1, Ward 3, Jinnah Postgraduate Medical Centre, Karachi, from November 2020 to April 2021. After approval was received from the Ethical Review Board (NO.F.2-81/2020-GENL/44820/JPMC) dated July 22, 2020, data collection commenced.

All patients were tested for coronavirus disease 2019 (COVID-19) via nasal swab, and only those with negative PCR results in the last 48 hours were included. Pregnant females, any allergy to the local anesthetic drug, patients with weight less than 30 kilograms, those with known diabetes and having uncontrolled sugar levels with a fasting blood glucose level of more than equal to 110mg/dl, known hypertensive with a systolic blood pressure of more than equal to 140 mmHg, those with having ejection fraction of less than equal to 40% with ischemic heart disease and forced expiratory volume 1 to be less than equal to 70% of the normal in patients with pulmonary disease as per history and clinical record were excluded. History of long-term usage of the oral pain killers for more than one month, those with blood loss of more than one blood volume within 24 hours around 70ml/kg, and excess continuous blood collection into the drains were not included in the study.

Patients were planned for MRM after detailed history, clinical examination, ultrasound or mammographic findings, and biopsy. Those patients who had carcinoma on histopathology and fulfilled the inclusion criteria underwent MRM. They were preoperatively randomized into two designated groups by lottery method. Group B (bupivacaine receiving patients) received 40 ml of injection bupivacaine 0.25%, 20 ml through each axillary and chest wall drain, and Group C (control) had no instillation.

Patients were taught before the operation about the visual analog scale (VAS: 10 cm scale) to assess their postoperative pain. All routine investigations were taken strictly before surgery. Once in the operating room, IV was secured with an 18-gauge cannula sited under aseptic measures in the non-dominant hand, and 500 ml of Ringer's lactate preload was done. All standard monitoring, i.e., ECG, non-invasive blood pressure (NIBP), and arterial blood gas (ABG), was obtained throughout. Standard general anesthesia was induced with injection propofol with a dosage of 2mg/kg, and the opioid (injection fentanyl with a dosage of 2μg/kg) was administered. On completion of the surgical procedure, two drains were placed. One drain was placed in the axillary region, and the other one in the chest wall over the pectoral muscle was placed before closing the incision.

Group B patients were the study group who received bupivacaine. The drains were kept nonfunctional by clamping them for a short 10 min time and released afterward to allow the bupivacaine to run from positive pressure into the negative pressure redivac drain. Neuromuscular blocking agents neostigmine and glycopyrrolate were used to reverse the anesthesia. Post-extubation patients have transferred to the recovery room for post anesthesia vital monitoring and pain control management. Duration of analgesia was calculated in hours starting when the patient was received in the recovery room till the patient felt the pain of score >3 according to the Visual Analogue Pain Score chart (VAS). Pain score at 0 hours was noted after extubation and subsequently every fourth hour by a trained resident for 24 hours. Similarly, Group C patients' span of analgesia was recorded without the instillation of a drug through the VAS scoring chart. All the data was noted and recorded in the attached proforma, along with the demographic details of the patient. All the general anesthesia was given by the same consultant. All MRMs were performed by the same surgeon and team to eliminate biases, and all confounding variables were controlled through exclusion criteria.

SPSS version 25 (IBM Corp., Armonk, NY, USA) was used to analyze and compute the collected data. A sample size of 60 was calculated with 96% power of the test on a 95% confidence interval while taking the expected mean duration of postoperative pain relief in patients undergoing MRM to be 14.6±9.6 h vs 4.3±5.2 hours in the bupivacaine and control group respectively [10]. Numerical variables, i.e. age, duration of surgery, and postoperative duration of analgesia were presented by mean±SD and range. An independent sample t-test was applied for comparison of the mean span of analgesia between both the groups taking a p-value ≤0.05 as statistically significant. The categorical variable, i.e., American Society of Anesthesiologists (ASA) class, was presented as frequency and percentage. Data was stratified for age, duration of surgery, and ASA class. Post-stratification T-test was applied, taking a p-value of ≤0.05 as statistically significant. After informed consent, 60 female patients aged between 45 to 60 years with the ASA Class I or II undergoing unilateral MRM with axillary lymph node dissection were included in this study.

## Results

**Figure 1:**
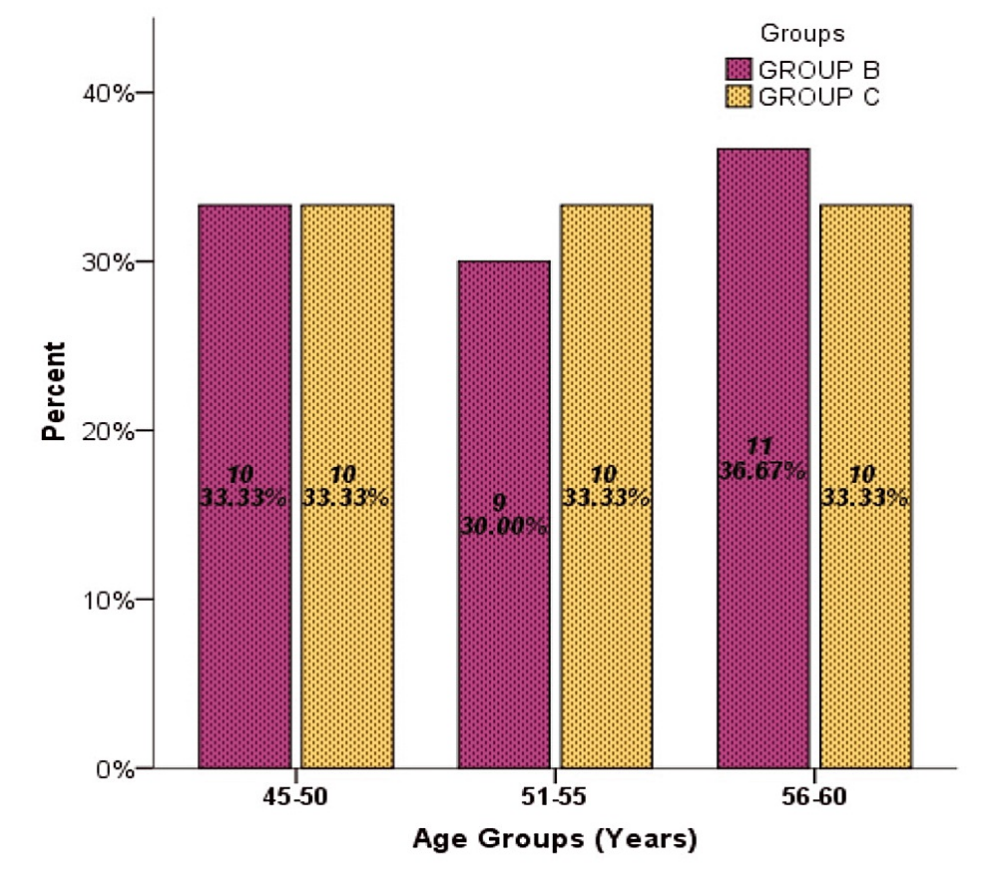
Age Distribution According to Groups Among a Sample Size of 60

**Figure 2:**
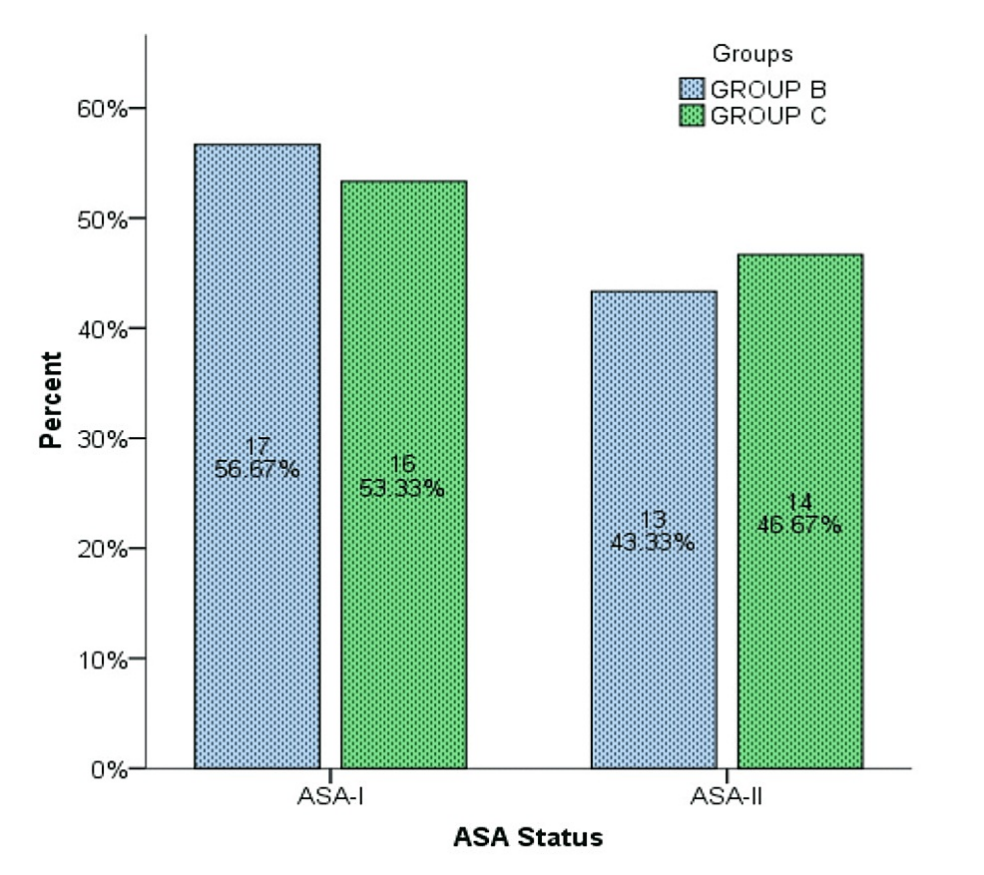
American Society of Anesthesiologists (ASA) Status of 60 Patients Between Group B and Group C

A total of 60 women who underwent MRM were randomly assigned into two groups. In Group B 30 women received 40 ml of 0.25% injection bupivacaine, 20 ml through each axillary and chest wall drain, and 30 women in Group C received no instillation. The average age distribution was 52.48±4.76 years (Figure 1). ASA-I was observed in 33 (55%) patients, and ASA-II was in 27 (45%) (Figure 2). The longest surgery duration was recorded at 95 min while the minimum duration of surgery was recorded at 40 min with a mean time period of surgery in Group C was recorded higher 70.27 min in comparison to Group B which was 63.70 min. The mean period of pain relief was considerably higher in Group B compared to Group C, i.e. 10.93±1.84 vs 5.03±1.35 hours. Stratification analysis of comparison of the mean age group (45-50, 51-55, and 56-60 years), duration of surgery, and duration of analgesia with groups B and C were computed through an independent sample t-test. Duration of analgesia showed strong evidence of a difference in the mean when compared with groups B and C (t = 14.17, df = 58, p-value = 0.000). Age group and duration of surgery showed no strong relation in comparison to means. The Independent Samples Test was used for testing the null hypothesis that the variances in the two groups are equal (Table 1).

**Table 1:** Comparison of mean duration of analgesia between groups in patients undergoing MRM stratified with age, ASA Class I and II, and duration of surgery. t-test for Equality of Means ASA: American Society of Anesthesiologists, MRM: modified radical mastectomy

		t	df	Sig. (2-tailed)	Mean Difference	Std. Error Difference	95% Confidence Interval of the Difference	
							Lower	Upper
Age Group	Equal variances assumed	.154	58	.878	.033	.217	-.401	.468
	Equal variances not assumed	.154	57.968	.878	.033	.217	-.401	.468
DSurgery	Equal variances assumed	-1.275	58	.208	-6.567	5.152	-16.879	3.746
	Equal variances not assumed	-1.275	57.999	.208	-6.567	5.152	-16.879	3.746
DAnalgasia	Equal variances assumed	14.170	58	.000	5.900	.416	5.067	6.733
	Equal variances not assumed	14.170	53.279	.000	5.900	.416	5.065	6.735

**Figure 3:**
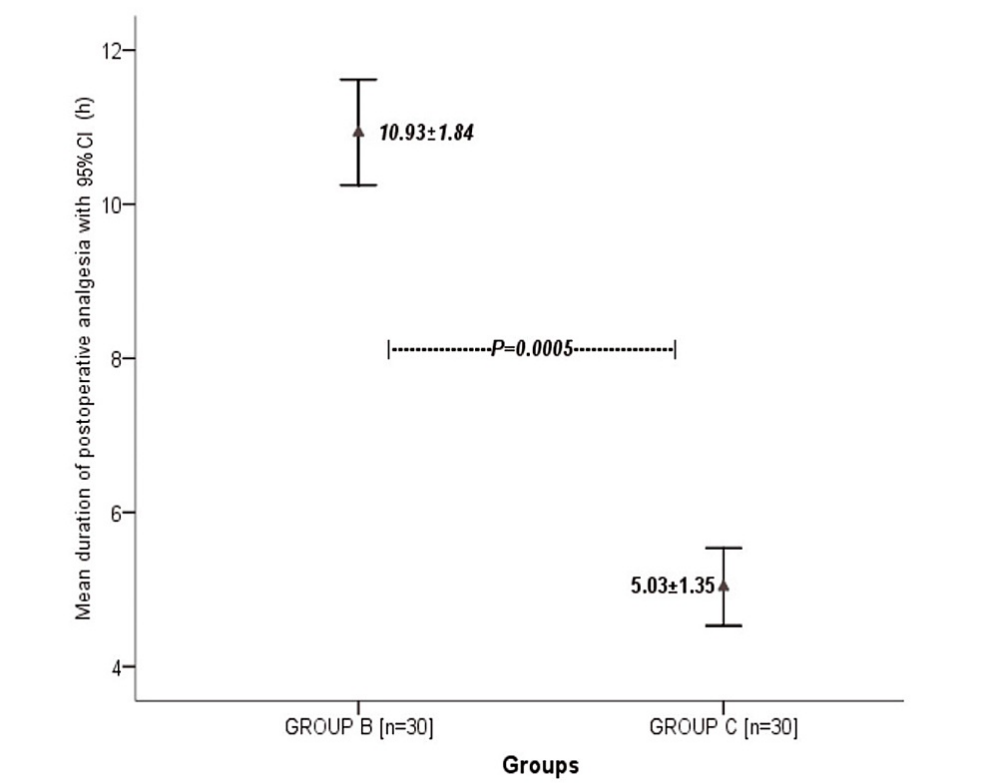
Graph Representation of a Comparison of Mean Duration of Analgesia Between Groups in Patients Undergoing Modified Radical Mastectomy

In particular, in this study, the test suggests that there is a marked difference in the duration of analgesia within Group B and Group C with a p-value below 0.005 (Figure 3). 

## Discussion

MRM is a procedure that involves extensive dissection of the chest wall, removing entire breast tissue with the overlying skin and nipple along with axillary clearance. It comes as no surprise that such extensive tissue dissection leads to both intraoperative and postoperative pain of similar magnitude [11]. The pain, if not treated, leads to adverse clinical consequences in terms of both halted postoperative recovery and complications due to decrease mobility. Thus the alleviation of pain is one of the pillars critical to patient care. Perioperative analgesia helps in minimizing the surgical stress given to the patient and decreases the abrupt changes in both physiological and immunological responses. This leads to patient satisfaction and early rehabilitation. It's a well-known fact that poor analgesic control is one of the common reasons for long-term post-mastectomy pain.

As per the WHO analgesic ladder guidelines for pain management [12], multiple routes can be taken for good analgesic control ranging from patient-controlled analgesia to oral plus parenteral NSAIDs, opioids, co-analgesics (anti-depressants), and local anesthesia. Other measures already used for post-MRM pain control include local anesthesia infiltration, infusion, intercostal blocks, paravertebral block, thoracic epidural anesthesia, inter-pleural block, and ultrasound-guided inter-fascial block are also used. Several techniques are available for postoperative wound site-local anesthesia application. Wound instillation with local anesthesia is not only simpler in terms of application and technicality but also has no added risk associated with its infiltration, including tension pneumothorax [13].

Multiple studies conducted around the globe have shown the increased requirement of opioids for pain control post mastectomy, especially in those patients who underwent bilateral mastectomy and then early breast reconstructive procedures, when compared with other breast surgeries [14-15]. Although an excellent analgesic, excessive and irrational use of opioids has a wide spectrum of side effects, ranging from nausea to respiratory depression [16]. The possible abuse and high potency for dependence further limit its benefit. Regional nerve and field blocks are an efficient means for getting a good analgesia post MRM but require expertise for its application. However, the results are achieved with variable success and have the possibility of creating iatrogenic pneumothorax during infiltration [5]. There are chances of spread of local anesthetics, up to 70% of the infiltrated content, from paravertebral space into the epidural space [17].

In a prospective RCT, the resulting instillation of 0.25% injection bupivacaine via surgical drains inpatient undergoing MRM showed better analgesia in the postoperative period compared to the control group with no intervention done. We found that the mean time period of pain relief was much higher in Group B when compared to Group C (10.93±1.84 hours vs 5.03±1.35), supporting the hypothesis that there is a difference in the meantime period of analgesia in patients undergoing MRM with wound treated with bupivacaine. Our results are supported by Chhatrapati et al., with the duration of analgesia found to be much longer in their bupivacaine group with a p-value of <0.0001 [9]. Moreover, our study was better as we used 0.25% compared to the 0.125% strength of local anesthetic used in the former article.

Alhussini et al. found that there was a marked decrease in VAS in the first 24 hours postoperatively in the bupivacaine group. Subsequently, the use of the analgesic drug was also reduced [18]. Likewise, Chhatrapati et al. studied VAS in the postoperative period at one hour, 10 hours, and 12 hours and was found to show good pain relief in the bupivacaine group [9]. This supports our research as cumulative rescue analgesic consumption for pain relief in the first 24 hours with a pain score ≥6, which was significantly low in the bupivacaine group.

Another study by Wang et al. used a similar technique of infiltrating ropivacaine 4, long-acting local anesthesia with comparable anesthetic effect to bupivacaine but better safety profile [19], in the postoperative drains used in mastectomy to control pain. Patients with ropivacaine intervention showed a significant reduction in pain was improved quality of life.

Patel et al. resulted from the indifference of the ropivacaine group and control of total tramadol consumption (P < 0.0001) with the duration of analgesia (P < 0.0001) [20]. They studied VAS at six hours, 12 hours, and 24 hours after MRM and it was remarkably higher in the control group compared to bupivacaine. In either group, none of the patients developed any side effects with no excess collection of blood into the drains, which was unpredictable action of the drug. No case of local anesthetic toxicity was reported.

Study limitations

The occurrence of seroma and hematoma formation in the surgical wound, postoperative hemodynamic monitoring, infection in the wound, and patient body mass index were some of our limitations. We only compared with control and not any other local anesthetic drug. The duration of analgesia could be further prolonged if the continuous analgesic drug were infused through surgical drains. In this regard, new studies should be conducted using infusion catheters with different local anesthetics agents. There was no record of chronic pain on follow-up after discharge, and further work can be done to look for possible long-term effects of wound instillation with local anesthesia.

## Conclusions

Instillation of the wound with injection bupivacaine via surgical drains after MRM with axillary lymph node dissection offers better postoperative pain relief and decreases the need for oral and intravenous pain killer requirements. Moreover, patients perform well after surgery and recover rapidly postoperatively without the need for costly drugs. Thus, this technique should be used routinely in MRM surgeries.
